# A Case of Multiple Myeloma with Metachronous Chronic Myeloid Leukemia Treated Successfully with Bortezomib, Dexamethasone, and Dasatinib

**DOI:** 10.1155/2014/962526

**Published:** 2014-12-02

**Authors:** Samer Alsidawi, Abhimanyu Ghose, Julianne Qualtieri, Neetu Radhakrishnan

**Affiliations:** ^1^Department of Internal Medicine, University of Cincinnati, Cincinnati, OH 45267, USA; ^2^Division of Hematology-Oncology, Department of Medicine, University of Cincinnati, Cincinnati, OH 45267, USA; ^3^Department of Pathology, University of Cincinnati, Cincinnati, OH 45267, USA

## Abstract

The coexistence of multiple myeloma and chronic myeloid leukemia in a single patient is a very rare event that has been reported very infrequently in the literature. We report a case of a patient who developed chronic myeloid leukemia four years after his diagnosis with multiple myeloma. Historically, no link between the two malignancies has been identified. This synchronous existence complicates the treatment plan for these patients, and there is a lack of evidence on the best therapeutic approach. Our patient was successfully treated with a combination of bortezomib, dexamethasone, and dasatinib, which he tolerated well for eleven months until he eventually succumbed to cardiac complications and pulmonary hypertension leading to his death.

## 1. Introduction

Multiple myeloma (MM) is a plasma cell dyscrasia characterized by an uncontrolled proliferation of a single plasma cell clone leading to overproduction of a monoclonal immunoglobulin. MM is an overall uncommon malignancy accounting for approximately 1% of all cancers in the Unites States [1]. Chronic myeloid leukemia (CML) is a myeloproliferative disorder characterized by uncontrolled proliferation of mature granulocytes. CML is associated with the fusion of two genes:* BCR* (on chromosome 22) and* ABL1* (on chromosome 9) resulting in the* BCR-ABL1* fusion gene which gives rise to an abnormal chromosome 22 known as the Philadelphia chromosome which is closely linked to the pathogenesis of this malignancy. CML is also considered an uncommon malignancy with an annual incidence of 1 to 2 cases per 100.000 [2]. About 10 to 15 percent of patients with CML initially present in the accelerated phase or blast phase of the disease which resembles an acute leukemia. The cooccurrence of MM and CML in one patient is an extremely rare incident that has been reported in less than 20 cases in the literature and their simultaneous presence or management is not yet fully understood. Here we present the case of a patient who achieved good response to his MM treatment but presented with CML four years after his MM diagnosis which further complicated his treatment plan.

## 2. Case Presentation

**Figure 1:**
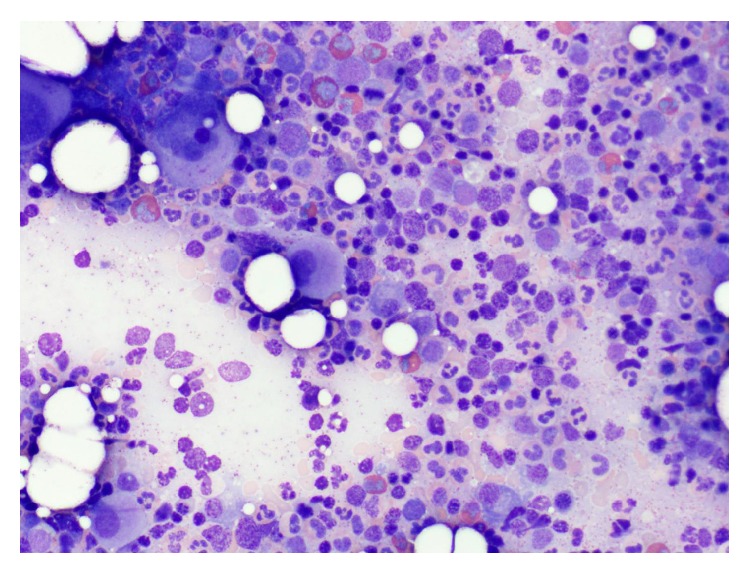
Bone marrow aspirate showing a hypercellular marrow with trilineage hematopoiesis and increased myeloid cell series (M : E ratio 4 : 1) with eosinophilia and plasmacytosis (approximately 15 to 20%).

**Figure 2:**
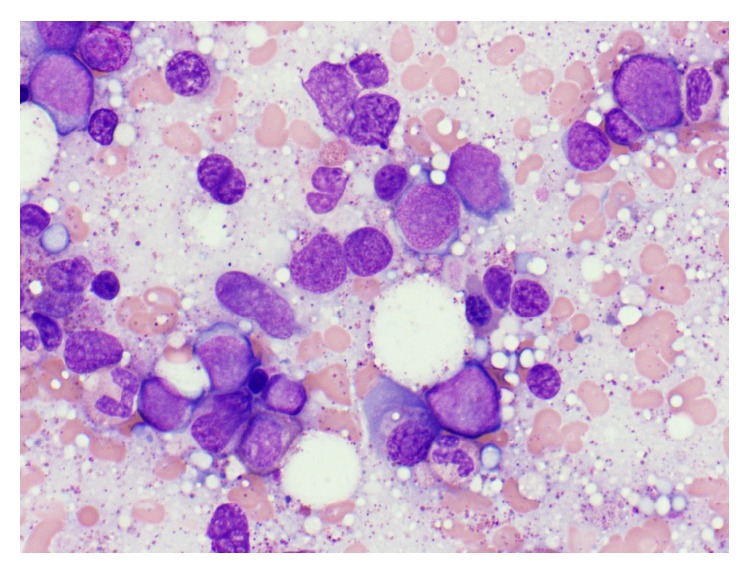
Bone marrow with markedly increased population of cells >20%, morphologically consistent with blasts.

A 60-year-old African American male with beta-thalassemia minor, diabetes mellitus, and hypertension was diagnosed with IgG Kappa multiple myeloma (MM) in 2008 after a minor mechanical fall resulted in a right femur fracture. His X-rays showed multiple lytic lesions of the right femur. Labs at diagnosis were significant for microcytic anemia with a hemoglobin of 12.3 g/dL and a mean corpuscular volume (MCV) of 67 fL, normal white blood cells (WBC) and platelet counts, an M-spike of 3.9 g/dL on his serum protein electrophoresis (SPEP) with the immunofixation significant for a monoclonal IgG Kappa, and a high Kappa/Lambda ratio. There was 70% involvement of the bone marrow with plasma cells and the cytogenetic analysis was significant for male karyotype with trisomy of chromosome 3 and deletion of Y chromosome in 4 out of 20 cells. The patient was treated with radiation to his femur after an orthopedic procedure and this was followed by five cycles of lenalidomide 25 mg daily on days 1–21 and dexamethasone 40 mg daily on days 1, 8, 15, and 22 every 4 weeks followed by bortezomib 1.3 mg/m^2^ on days 1, 4, 8, and 11 every 3 weeks. The patient achieved stable disease with this regimen and he received no further treatment for almost four years. Meanwhile, he was being followed up regularly with clinical and laboratory assessments, serial serum protein electrophoresis with immunofixation, and free light chains. In 2012, four years after his initial diagnosis and treatment, the patient was found to have a relapse of his MM with an increase in his M-spike to >2 g/dL. Labs showed hemoglobin of 9.5 g/dL, WBC of 11.2 × 10^3^/mm^3^ with a normal differential except for slightly elevated eosinophils, and platelet count of 509 × 10^3^/mm^3^. Skeletal survey showed increase in his myeloma lytic bone lesions. A bone marrow aspirate and biopsy was done which demonstrated a hypercellular marrow with trilineage hematopoiesis, increased myeloid cell series (M : E ratio 4 : 1) with eosinophilia, and 15–20% monoclonal plasmacytosis based on CD138 immunostaining (Figure 1). The cytogenetics showed 46 XY t(9;22) (q34;q11.2) in 8 out of 15 cells and the fluorescent in situ hybridization (FISH) was consistent with BCR/ABL translocation in 72.8% of cells. Interestingly, no trisomy of chromosome 3 or deletion of Y chromosome was detected in the cells examined—which could be explained by their low levels and the small number of metaphases examined. It was decided to control the patient's myeloma first prior to starting a tyrosine kinase inhibitor (TKI) for his CML as there was evidence of an aggressive myeloma progression with the sudden increase in his bony lesions, while his WBC was normal at the time. He was started again on lenalidomide 25 mg every 3 weeks and dexamethasone 20 mg weekly. Three months later, the patient presented with symptoms of fatigue, malaise, abdominal discomfort, and generalized weakness. The M spike was 1.8 g/dL, WBC count 42.7 10^3^/mm^3^, and platelet count of 2472 10^3^/mm^3^. There was an increased number of myelocytes, metamyelocytes, bands, mature neutrophils with rare blasts (approximately 1% of total cells), and increased basophils (4.2%). He underwent a bone marrow aspirate and biopsy that showed a hypercellular bone marrow with significantly increased myeloid progenitor population with a blast count of 37% in background of a myeloproliferative disorder, favoring transformation to acute myeloid leukemia (Figure 2). Unfortunately, the initial bone marrow biopsy from his MM diagnosis was not available to be examined for CML. A diagnosis of chronic myeloid leukemia in blast phase was made and cytoreductive treatment was started with hydration and hydroxyurea (up to 4 g/days) and subsequently he was started on dasatinib 140 mg daily. The patient reached major molecular response four months after treatment and complete molecular response four months after that. He was kept on maintenance treatment with dasatinib 100 mg daily. The treatment for the MM with lenalidomide was stopped when the patient presented in blast crisis. In 3 months, the patient had a quick progression of multiple myeloma with an increase of his M-spike to 2.5 g/dL and new diffuse lytic bone lesions. Treatment with weekly bortezomib 0.7 mg/m^2^ which was later increased to 1.3 mg/m^2^ weekly and dexamethasone 20 mg on days 1, 4, 8, and 11 of a 21-day cycle was restarted, concurrent with dasatinib. The patient tolerated the treatment combination of bortezomib and dasatinib well. He continued to show complete molecular response of his CML and he initially showed a good response of his MM as his M-spike stabilized around 1.9 g/dL transiently before increasing back to 2.3 g/dL again. One year from the start of dasatinib, the patient had an acute myocardial infarction. Dasatinib was continued while bortezomib and dexamethasone were discontinued which resulted in an improvement in his anemia while his M-spike remained stable. About five months later, he was admitted with pulmonary artery hypertension (PAH) with a mean pulmonary arterial pressure of 50–55 mm Hg on right heart catheterization. It was assumed that this was due to either the dasatinib or a diet pill that he admitted to taking over the counter or due to a combination of his comorbidities. Although dasatinib was stopped, he eventually succumbed to respiratory failure due to PAH 18 months after his CML diagnosis.

## 3. Discussion

The cooccurrence of MM and CML is a very rare entity that has been reported in less than twenty cases in the literature. There are only four reported cases where the diagnosis of MM preceded the diagnosis of CML [3–6]. Many of the reported cases in which the diagnosis of MM followed a long treatment course of CML [7–11] with the tyrosine kinase inhibitor, imatinib, suggested a link between this treatment and the development of MM. However, almost half of the reported cases of these two entities coexisting in a single patient are of patients who either developed MM first or were diagnosed with the two malignancies simultaneously [12–17]. This makes a link between imatinib and the development of MM very unlikely. Similarly, MM treatment does not seem to lead to the development of CML as the reported patients were treated for MM using regimens consisting of multiple different and unrelated agents. There is no evidence in the literature to suggest that either lenalidomide or bortezomib can lead to the development of CML. Lenalidomide is now known to be a risk factor for secondary malignancies, but it is usually nonmelanoma skin cancers and myelodysplastic syndromes [18]. The finding of Philadelphia chromosome in the bone marrow is an indicator of a myeloproliferative disease; however, there are reported cases of patients with MM with this translocation [19, 20]. The significance of such finding is unknown.

In the case presented above, we show that the combination of bortezomib and dasatinib was well tolerated. This represents the first case in which a patient with MM and CML received concurrent treatment with bortezomib, dexamethasone, and dasatinib. Pulmonary arterial hypertension is a known complication of dasatinib and is commonly seen after 8 to 48 months of treatment [21–24]. Although less common, pulmonary arterial hypertension has been reported with bortezomib as well [25, 26]. It was unclear if dasatinib was the only culprit in our patient's pulmonary hypertension as he also sustained a cardiac event and he later admitted to taking an unknown “diet pill” which might have all contributed to developing pulmonary hypertension. Most cases of pulmonary hypertension after dasatinib described near complete regression of this side effect after cessation of the drug. However, there are cases of refractory pulmonary hypertension that required treatment despite the withdrawal of dasatinib [27].

## 4. Conclusion

The cooccurrence of MM and CML is an extremely rare entity that creates dilemmas in the management of these two separate malignancies. We present our experience in a patient whose treatment of MM was complicated by the development of CML that quickly progressed into the blast phase. We found that the combination of bortezomib, dexamethasone, and dasatinib was well tolerated; however, special attention needs to be paid to the individual side effects of these medications.
